# Approximate analytical prediction on elastic properties of Diamond structures with varying porosities and orientations

**DOI:** 10.3389/fbioe.2025.1626104

**Published:** 2026-01-05

**Authors:** Hao Wang, Yongtao Lyu, Jian Jiang, Feihu Zhao, Sergei Bosiakov, Hanxing Zhu

**Affiliations:** 1 Department of Spinal Surgery, Central Hospital of Dalian University of Technology, Dalian University of Technology, Dalian, China; 2 School of Mechanics and Aerospace Engineering, Dalian University of Technology, Dalian, China; 3 DUT-BSU Joint Institute, Dalian University of Technology, Dalian, China; 4 Department of Biomedical Engineering, Zienkiewicz Institute for Modelling, Data & AI, Faculty of Science and Engineering, Swansea University, Swansea, United Kingdom; 5 Faculty of Mechanics and Mathematics, Belarusian State University, Minsk, Belarus; 6 School of Engineering, Cardiff University, Cardiff, United Kingdom

**Keywords:** triply periodic minimal surface, diamond structure, approximate analytical approach, finite element method, theory of elasticity, effective elastic modulus

## Abstract

**Introduction:**

Bone scaffolds are widely used for repairing bone defects. As a biomimetic structure for bone scaffolds, the triply periodic minimal surface (TPMS) structure is an ideal choice. To evaluate/characterize the mechanical properties of TPMS structures, multiple methods (e.g., via experiment or theoretical analysis) can be used. Each method has its advantages and disadvantages. Using approximate analytical approach, the mechanical properties of structures can be predicted quickly and efficiently. Therefore, it is necessary to determine the applicable range to ensure that the calculated mechanical properties of TPMS structures with varying porosity and strut orientation are acceptable.

**Methods:**

In this paper, approximate analytical prediction of elastic properties of TPMS structures (i.e., Diamond) with varying porosities and strut orientations was investigated, and finite element (FE) method and theory of elasticity were compared with the approximate analytical approach. The ranges for porosity were from 70% to 90%. The ranges for orientation were defined by rotating the scaffold from 0° to 90° along the [100] and [110] directions, and from -30° to 90° along the [111] direction. Due to the cubic symmetry of Diamond structure, these angular ranges ensure that the structure is non-repeating and is comprehensively analyzed in all three directions. Additionally, experimental tests were performed to validate the feasibility of the non-experimental methods.

**Results:**

It was shown from the experimental validation that the results from non-experimental methods were acceptable at certain porosities and orientations. The FE method, which is commonly used and a reliable approach, was utilized to represent the non-experimental methods and was compared with the experimental results. Therefore, the approximate analytical solutions and the results from elasticity theory were indirectly compared with experimental results. When the porosity of the structure was 85%, the approximate analytical solution showed differences of 17.65% relative to the FE result and 39.13% relative to the elasticity theory result. Therefore, the approximate analytical solution was considered acceptable at a higher porosity. The acceptable ranges of the porosity for applying the approximate analytical approach were higher than 85% in the [001] and [110] directions, and higher than 90% in the [111] direction. At the same structural porosity, in the (100) plane, the predicted results were acceptable when the structural orientation was close to 0° or 90°. In the (110) plane, the predicted results were acceptable when the structural orientation was close to 0°. In the (111) plane, whether the predicted results can be accepted or not was basically independent of the structural orientation but was dependent on the porosity of the structure. The planes of (100), (110) and (111) are defined as the planes perpendicular to the directions of [100], [110] and [111], respectively.

**Discussion:**

Data in the present study provide valuable guidance on applying the approximate analytical approach to efficiently predict the mechanical properties of TPMS structures prior to performing formal calculations and experiments.

## 1 Introduction

Bone scaffolds are widely used for repairing bone defects. As a biomimetic structure for bone scaffolds, the triply periodic minimal surface (TPMS) structure is an ideal choice. Under daily physiological loading, efficient load transmission and optimal stress distribution are exhibited on the bone tissue (Huiskes et al., 2000). Therefore, sufficiently high stiffness and strength of bone tissue were ensured under the lowest possible bone mass (Bobbert et al., 2017). Topologically designed bone substitutes are implanted to mitigate pain and reconstruct bone in patients with bone defects (Wang et al., 2016). With the development of additive manufacturing technology, TPMS structures with different topologies have been custom-designed and printed (Yu et al., 2019). The TPMS structure is porous, similar to cancellous bone, and exhibits excellent mechanical properties. Moreover, a suitable environment for osteoblast lineage and tissue growth and facilitated vascularization can be provided using TPMS structures with high permeability and sufficient surface area (Karageorgiou and Kaplan, 2005). Osseointegration, long-term stability, and longevity are mandatory considerations for bone implants (Wang et al., 2016). However, TPMS structures with different topologies exhibit different mechanical properties due to multiple factors, including porosity and orientation (Yang et al., 2019; Qureshi et al., 2022; Cai et al., 2019). Therefore, it is important to utilize appropriate methods to evaluate the properties of TPMS structures.

There are various methods for evaluating the mechanical properties of TPMS structures. To investigate the mechanical properties of the TPMS structure, various methods were used. Yang et al. (2019) utilized an analytical approach, the finite element (FE) method, and experiments to investigate the mechanical response of gyroid structures with different structural volume fractions and strut orientations. It was shown that the analytical solution is reasonable at volume fractions below 20%. Cai et al. (2019) designed TPMS models and then fabricated them using 3D printing to explore the effects of different porosities on the mechanical properties of the structures. There is a specific relationship between porosity and Young’s modulus of TPMS structures. Kang et al. (2020) developed a new numerical method based on the generalized Hooke’s law to investigate four porous microstructures, qualitatively and quantitatively evaluating the mechanical anisotropy between porous structures and host bone. Viet et al. (2022) investigated the mechanical properties of four TPMS structures using FE simulations and experimental tests. The characteristics of Young’s modulus, shear modulus, and Poisson’s ratio revealed significant differences between the solid and sheet types of TPMS structures. Rezapourian et al. (2023) experimentally and numerically evaluated the Ti6Al4V Split-P TPMS structures produced by selective laser melting (SLM). It was demonstrated that mechanical properties close to those of cancellous and cortical bone could be achieved using the Ti6Al4V Split-P lattices with the highest surface area and surface area-to-volume ratios. The TPMS scaffolds with multi-functional pores were investigated in our previous work (Jiang et al., 2024). This structural optimization mitigated stress shielding and improved mass transport capacity compared to original TPMS structures. Therefore, the mechanical properties of TPMS structures can be evaluated and characterized using different methods.

There are different advantages and disadvantages to these methods. The FE method can be used accurately, but a high computational cost should not be ignored in modeling and simulation. Theoretical calculation is relatively efficient, but lower accuracy of the results can be obtained due to the complex derivations of the equations and several assumptions. The experimental test is the most reliable approach, but it is time-consuming and expensive; some complex testing conditions are difficult to achieve. The approximate analytical approach is utilized due to its high efficiency in terms of numerical calculations. The approximate analytical approach first simplifies the original model into a model composed of struts and then determines the mechanical properties of the structure using existing mechanical formulas. This method can be chosen according to the conditions for specific need/focus (i.e., efficiency vs. accuracy). Therefore, it is important to understand the applicable range of this method to mechanically characterize TPMS scaffolds. However, investigating the applicable range of the approximate analytical method for the elastic properties of TPMS structures with varying structural porosities and orientations is rare.

In this study, approximate analytical prediction of the elastic properties of TPMS structures (i.e., diamond) with varying porosities and strut orientations was investigated, and the FE method and the theory of elasticity were compared with the approximate analytical approach. Furthermore, experimental testing was conducted to validate the feasibility of these non-experimental methods. The FE method, which is commonly used and well-established, was utilized to represent the non-experimental methods and was compared with the experimental results. Therefore, the approximate analytical solutions and the results from elasticity theory were indirectly compared with experimental results. The applicable range of the approximate analytical approach was analyzed by comparing the effective elastic moduli of the structures with different porosities and orientations. The approximate analytical approach can help engineers/researchers efficiently predict the mechanical properties of TPMS structures in the initial design and screening stage of porous biological scaffolds.

## 2 Materials and methods

**FIGURE 1:**
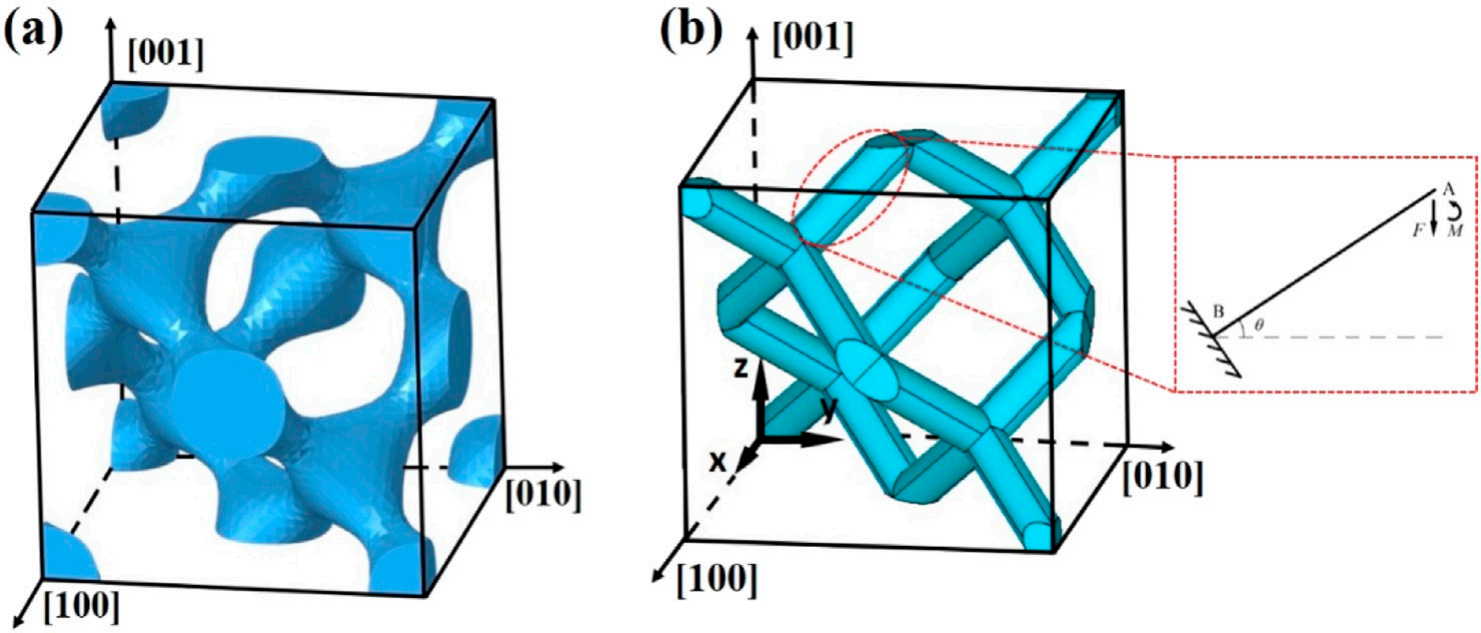
Schematic structure of Diamond. **(a)** Unit cell model. **(b)** Diamond simplified diagram and schematic diagram of forces on an inclined strut.

**FIGURE 2:**
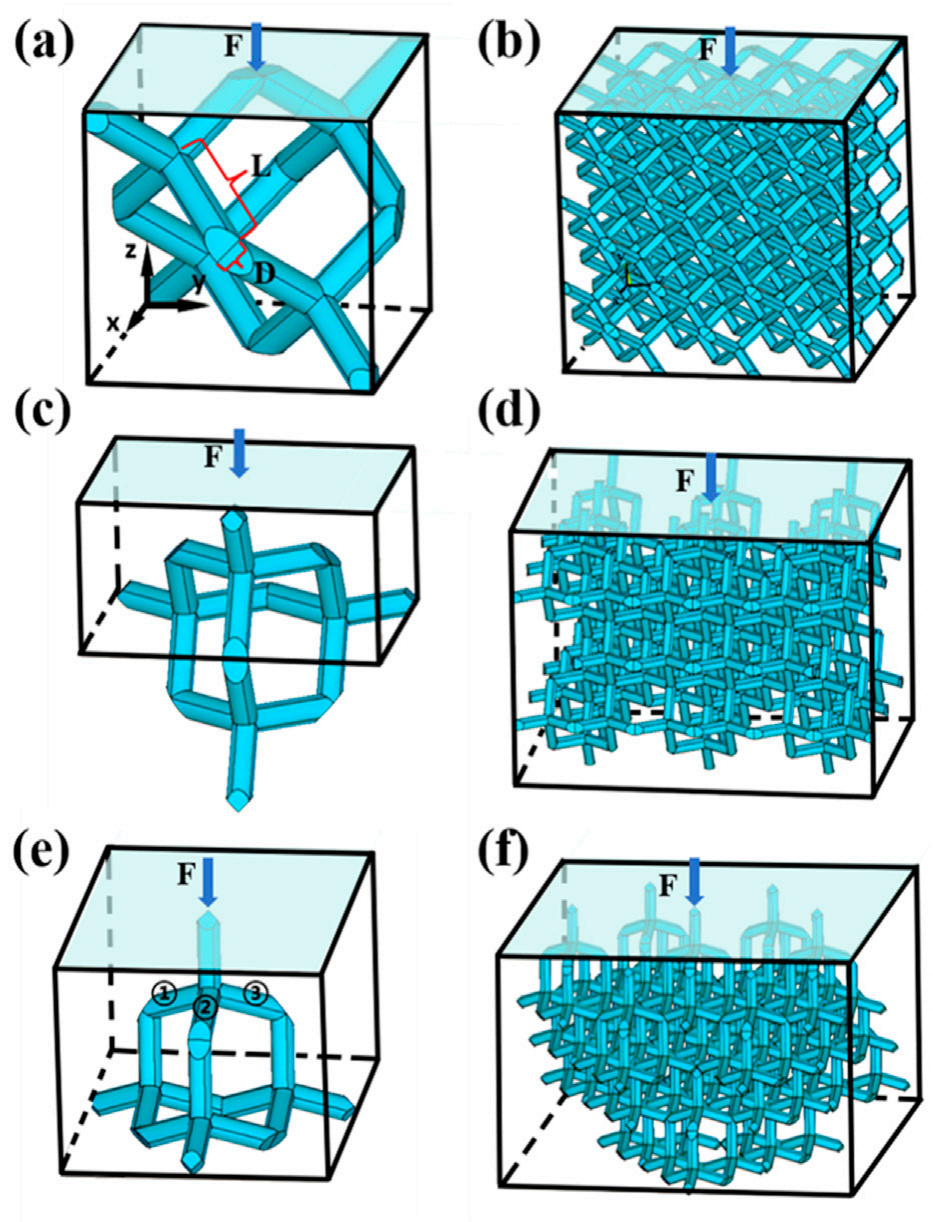
Schematic diagrams of **(a)** Simplified Diamond unit cell and **(b)** Diamond structure under 637 loading direction. **(c)** Simplified Diamond unit cell and **(d)** Diamond structure under loading direction. **(e)** Simplified Diamond unit cell and **(f)** Diamond structure under loading direction 639 [111]. All structures are loaded on their top face.

Using three non-experimental methods, namely, the approximate analytical approach, the FE method, and the theory of elasticity, an investigation was performed with the diamond TPMS structures as an example. The implicit mathematical expression of the diamond surface is presented in Equation 1:

$$\sin\left(X\right)\sin\left(Y\right)\sin\left(Z\right)+\sin\left(X\right)\cos\left(Y\right)\cos\left(Z\right)+\cos\left(X\right)\sin\left(Y\right)\cos\left(Z\right)+\cos\left(X\right)\cos\left(Y\right)\sin\left(Z\right)=t,$$ (1)

where X=2πx , Y=2πy , Z=2πz , and the function has a period of 1.0. *t* is a parameter that can be adjusted to obtain different shapes of surfaces, and therefore, bone scaffold models with different porosities can be obtained. The diamond scaffold with a solid volume fraction of 15% was modeled, as shown in Figure 1a. The diamond structure is rotationally symmetrical with a series of 90°, 120°, and 180° rotational symmetry axes. Their different rotational symmetries are shown in Figures 2a–f. The orientation dependence of the diamond structure in the effective elastic modulus is investigated for a series of directions in the (100), (010), and (001) planes as the loading directions. The effective elastic modulus is the Young’s modulus of structure under small deformations, characterizing its initial stiffness. The effective elastic modulus is one of the most important and commonly used mechanical properties of TPMS structures.

### 2.1 Prediction of the effective elastic modulus using the approximate analytical approach

#### 2.1.1 Simplification of the diamond structure

**FIGURE 3:**
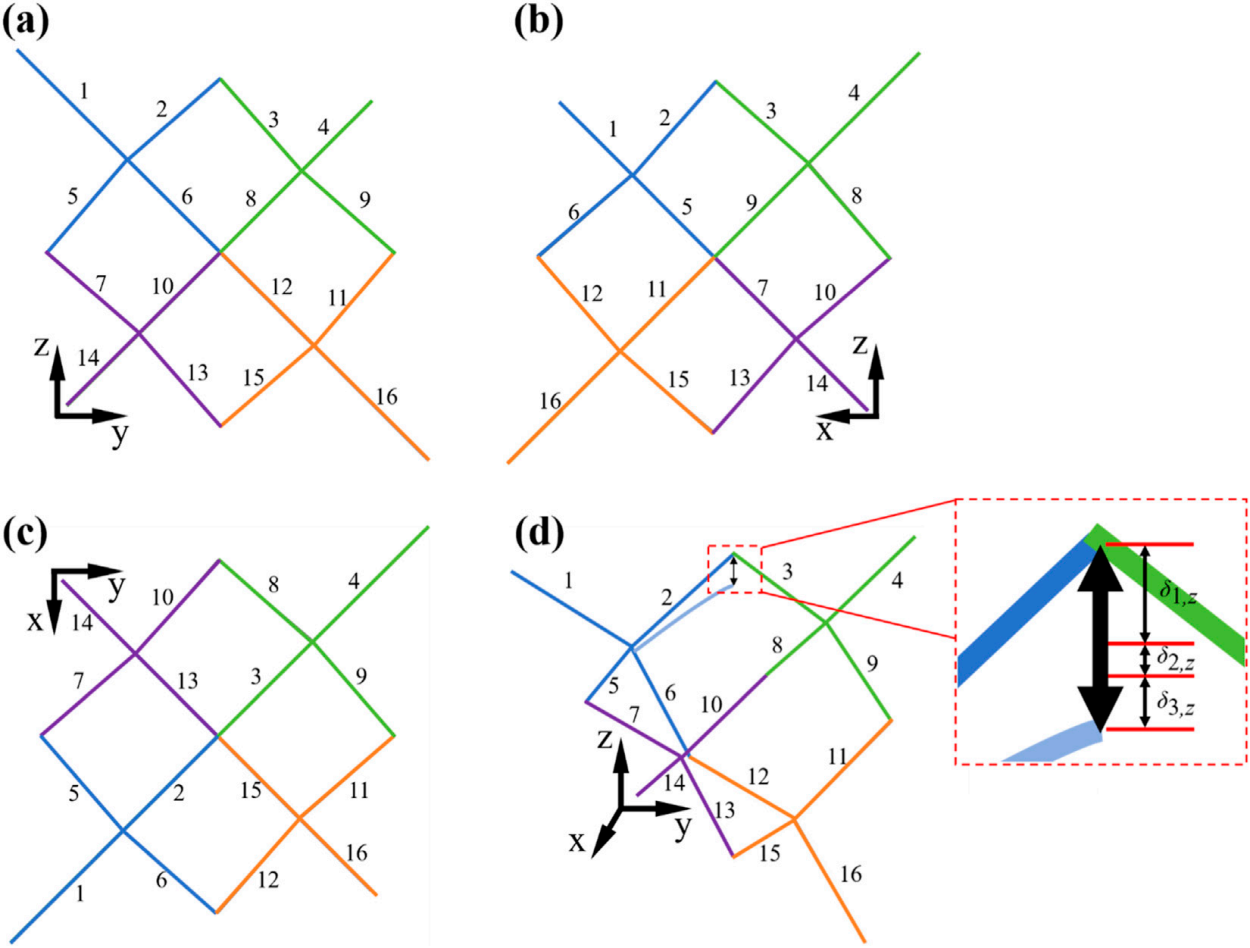
Topological relationship of the struts of Diamond unit cell with different views. **(a)** Front view. **(b)** Right view. **(c)** Top view. **(d)** Axonometric diagram and schematic diagram of deformation of an inclined strut in z-direction. All the struts in the unit cell are numbered in figures, the same number in different views represents the same strut. The positions of the struts of unit cell in different views can be clearly seen by numbering each strut and colors.

Although there are complex geometries in TPMS structures, the approximate analytical model can be used to efficiently predict the effective elastic moduli of the structures (Ahmadi et al., 2014; Hedayati et al., 2016; Wieding et al., 2014). The central axis of each strut was extracted, and the topological relationship of one diamond unit cell was obtained, as shown in Figure 3. The same number in different views represents the same strut. The positions of the unit cell struts in different views can be clearly observed by numbering and color-coding each strut.

There are 16 inclined struts in a diamond unit cell, as shown in Figure 3. *L* and *D* are the length and diameter of the inclined strut, respectively, as shown in Figure 2a. Defining the direction of the z-axis as the loading direction, the relationship among the length *L* of each inclined strut, the length a of the diamond unit cell, and the angle θ between the inclined strut and the horizontal plane is expressed in Equation 2:

$$a=2\sqrt{2}L\cos\theta =\frac{4\sqrt{3}}{3}L,\theta =35.26{}^{\circ}.$$ (2)

Based on the topological relationship, a simplified model of the diamond structure was proposed, and θ=35.26° in this case, as shown in Figure 1b. The struts with varying diameters were simplified to cylindrical struts of a uniform diameter, as shown in Figure 2a. It can be assumed that stiffness depends mainly on the smallest cross-section, where stress concentrations and failures commonly occur; therefore, the smallest diameter of the diamond strut can be taken as the minimum diameter of the original model (Yang et al., 2019). The topological relationships of the structure were preserved in the simplified model.

The volume fraction is defined as the ratio of the solid volume to the total volume of the corresponding bulk structure (Gibson and Ashby, 1997). The solid volume of all inclined struts in the diamond unit cell ( $V_{1}$ ) is expressed in Equation 3:

$$V_{1}=16\cdot \frac{\pi D^{2}}{4}\cdot L=4\pi D^{2}L,$$ (3)

and the volume of the cubic unit cell ( $V_{2}$ ) is expressed in Equation 4:

$$V_{2}={\;a}^{3}={\left(\frac{4\sqrt{3}}{3}L\right)}^{3}=\frac{64\sqrt{3}}{9}L^{3}.$$ (4)

The solid volume fraction *ρ* of the diamond structure can be obtained as presented in Equation 5:

$$\rho =\frac{V_{1}}{V_{2}}=\frac{4\pi D^{2}L}{\frac{64\sqrt{3}}{9}L^{3}}=\frac{3\sqrt{3}\pi D^{2}}{16L^{2}}.$$ (5)

#### 2.1.2 Effective elastic modulus in different orientations

The simplification of the geometric model not only preserves the topological relationships but also facilitates calculations. The solid material was designated as isotropic and linearly elastic. The effective elastic modulus of diamond structures can be calculated using the Euler–Bernoulli or the Timoshenko beam theory (Timoshenko and Goodierwrited, 1970; Young et al., 2003).

**FIGURE 4:**
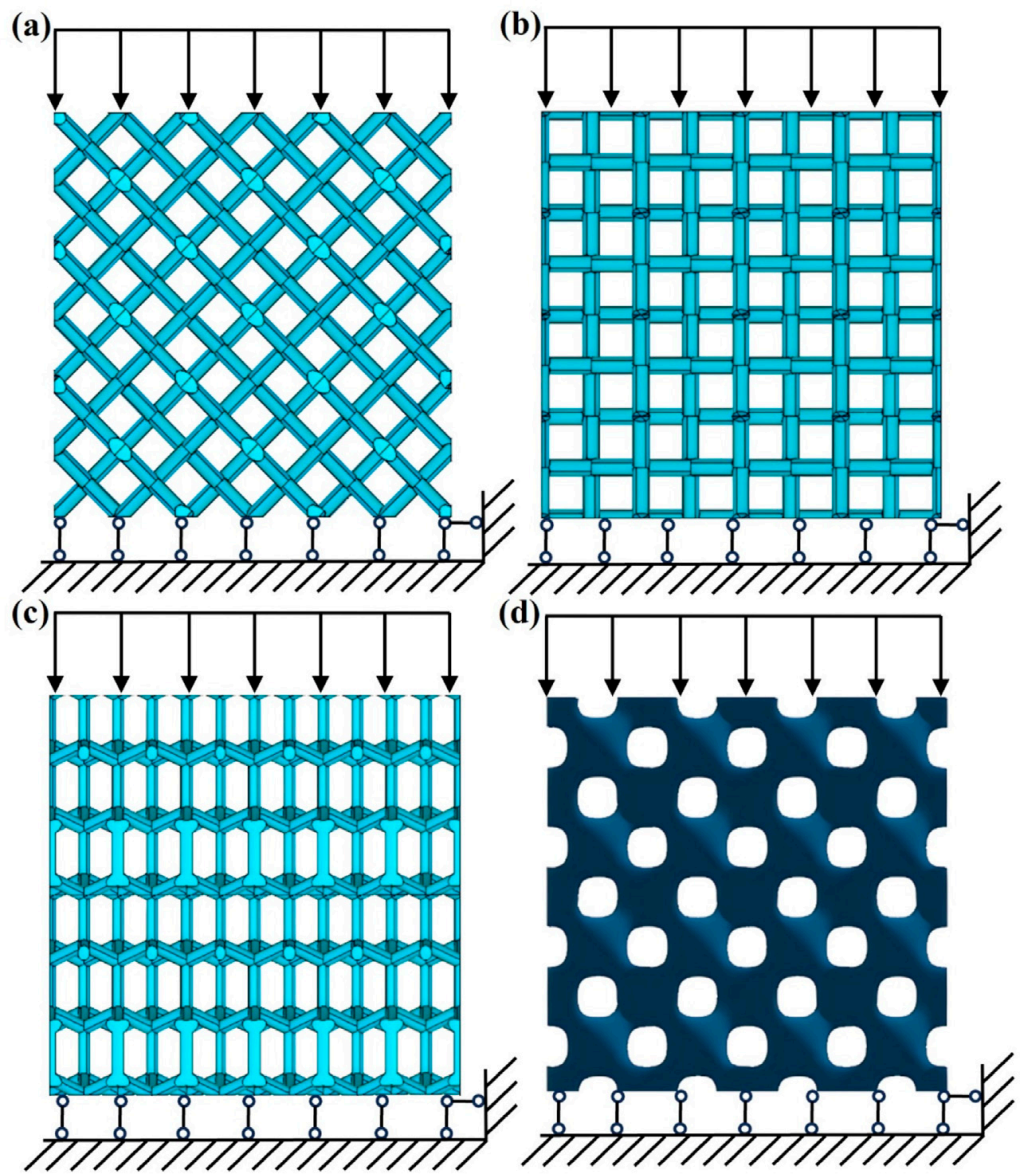
Schematic diagram of the Diamond structure and boundary conditions. **(a)** Loading direction [001]. **(b)** Loading direction [110]. **(c)** Loading direction [111]. **(d)** 4 × 4 × 4 Diamond lattice structure.

The uniaxial tensile or compressive stress along the z-direction was set to be *σ* in the diamond structure, as shown in Figure 4a. According to the structural symmetry, there was uniaxial tension or compression in the z-direction. The load transmitted to an arbitrary unit cell is $P={\;a}^{2}\sigma$ , which is carried by four inclined struts. The load (*F*) and bending moment (*M*) of each inclined strut can be obtained as shown in Equation 6 (Timoshenko and Goodierwrited, 1970; Young et al., 2003):

$$F=\frac{a^{2}\sigma }{4},M=\frac{FL\cos\theta }{2}.$$ (6)

The compression of a unit cell in the z-direction is four times the deformation of an inclined strut in the z-direction. The force on the inclined strut is shown in Figure 3d. The total deformation of each inclined strut consists of the bending deformation, shear deformation, and stretching deformation. The bending deflection can be obtained using the Euler–Bernoulli beam theory (Young et al., 2003). For a cantilever beam of length *l*, when a load $F_{1}$ and a bending moment $M_{1}$ are applied at the free end, the deflection of the cantilever beam can be calculated as presented in Equation 7 (Young et al., 2003):

$$w_{1}=\frac{F_{1}l^{3}}{3EI}-\frac{M_{1}l^{2}}{2EI},$$ (7)

where *E* and *I* denote the elastic modulus of the material and moment of inertia, respectively. In this study, the range of porosity of structures was 70%–90% (i.e., solid volume fraction = 30%–10%). The slenderness ratio was not large enough, so shear deformation cannot be ignored (Brassey et al., 2013; Turner and Burr, 1993). Therefore, considering the deflection caused by shear deformation, the final deflection calculated using the Timoshenko beam theory can be derived from Equation 8 (Timoshenko and Goodierwrited, 1970):

$$w_{2}=\frac{F_{1}l^{3}}{3EI}-\frac{M_{1}l^{2}}{2EI}+\frac{F_{1}l}{\kappa \;AG},$$ (8)

where *κ* is the Timoshenko shear coefficient, calculated for a solid cross-section according to Cowper (1966) ( $\kappa =\frac{6\left(1+\nu \right)}{7+6\nu }$ ); $G=\frac{E}{2\left(1+\nu \right)}$ is the shear modulus of the material; ν is Poisson’s ratio of the material; and A is the cross-section area of the inclined strut. The deformation of the inclined strut caused by the axial force $F_{2}$ is provided in Equation 9 (Timoshenko and Goodierwrited, 1970; Young et al., 2003):

$$w_{3}=\frac{F_{2}l}{EA}.$$ (9)

For an inclined strut in Figure 3d, deformation components in the *z*-direction caused by bending deformation, shear deformation, and axial deformation are presented in Equations 10–12, respectively:

$$\delta_{1,z}=\frac{F_{1}l^{3}}{3EI}-\frac{M_{1}l^{2}}{2EI}=\frac{FL^{3}\cos^{2}\theta }{12E_{s}I},$$ (10)

$$\delta_{2,z}=\frac{FL\cos^{2}\theta }{\kappa \;AG}=\frac{\left(7+6\nu \right)FLD^{2}\cos^{2}\theta }{48E_{s}I},$$ (11)

$$\delta_{3,z}=\frac{FL\sin^{2}\theta }{E_{s}A}=\frac{FLD^{2}\sin^{2}\theta }{16E_{s}I},$$ (12)

where $E_{s}$ is the elastic modulus of the solid material, $F_{1}=F/\cos\theta$ , $F_{2}=F\sin\theta$ , and $M_{1}=M.$ Therefore, the total deformation of an inclined strut in the *z*-direction can be derived by summing the three deformation components above, as shown in Equation 13:

$$\delta_{z}=\delta_{1,z}\;{+\delta }_{2,z}+\delta_{3,z}=\frac{FL^{3}\cos^{2}\theta }{48E_{s}I}\left[4+\left(7+6\nu +3\tan^{2}\theta \right){\left(\frac{D}{L}\right)}^{2}\right].$$ (13)

The compressive deformation of the unit cell δ was four times the deformation of a single inclined strut in the *z*-direction, as presented in Equation 14:

$${\delta =4\delta }_{z}=\frac{FL^{3}\cos^{2}\theta }{12E_{s}I}\left[4+\left(7+6\nu +3\tan^{2}\theta \right){\left(\frac{D}{L}\right)}^{2}\right].$$ (14)

For a unit cell, strain is defined as shown in Equation 15:

$$\varepsilon =\frac{\delta }{a}\cdot 100\%.$$ (15)

The Young’s modulus $E_{c}$ of the diamond structures can be calculated by dividing the stress by the strain, as presented in Equation 16:

$$E_{c}=\frac{\sigma }{\varepsilon }=\frac{4F}{\delta \;a}=\frac{\sqrt{3}F}{\delta \;L}=\frac{3\sqrt{3}\pi E_{s}D^{4}}{{16L}^{4}\cos^{2}\theta }\cdot \frac{1}{4+\left(7+6\nu +3\tan^{2}\theta \right){\left(\frac{D}{L}\right)}^{2}}.$$ (16)

Thus, the dimensionless Young’s modulus of the unit cell can be derived as

$$\frac{E_{c}}{E_{s}}=\frac{3\sqrt{3}\pi }{16\cos^{2}\theta }\cdot {\left(\frac{D}{L}\right)}^{4}\cdot \frac{1}{4+\left(7+6\nu +3\tan^{2}\theta \right){\left(\frac{D}{L}\right)}^{2}}.$$ (17)

Substituting the values of *θ* into Equation 17, the result of the dimensionless modulus of the diamond structure was obtained as shown in Equation 18:

$$\frac{E_{c}}{E_{s}}=\frac{9\sqrt{3}\pi }{16}\cdot {\left(\frac{D}{L}\right)}^{4}\cdot \frac{1}{8+21.08{\left(\frac{D}{L}\right)}^{2}}=\frac{6\rho^{2}}{3\sqrt{3}\pi +42.16\rho }.$$ (18)

In the [110] direction, the structure of the unit cell was used to calculate the Young’s modulus, and some sections of the inclined struts were hidden within the unit cell, as shown in Figure 2c. Due to structural symmetry, four inclined struts carry the load, as shown in Figure 4b. The result for the dimensionless modulus was obtained as presented in Equation 19:

$$\frac{E_{c}}{E_{s}}=\frac{9\sqrt{3}\pi }{16}\cdot {\left(\frac{D}{L}\right)}^{4}\cdot \frac{1}{4+15.04{\left(\frac{D}{L}\right)}^{2}}=\frac{12\rho^{2}}{3\sqrt{3}\pi +60.08\rho }.$$ (19)

The unit cell in the [111] direction was obtained, as shown in Figure 2e. As shown in Figures 2e, 3c, due to the structural symmetry, the load was carried by 8 vertical struts and transmitted to 24 inclined struts through the nodes. The blue arrow represents the applied mechanical loading, and the three inclined struts are marked, as shown in Figure 2e. The result for the dimensionless modulus was obtained as shown in Equation 20:

$$\frac{E_{c}}{E_{s}}=\frac{27\sqrt{3}\pi }{16}\cdot {\left(\frac{D}{L}\right)}^{4}\cdot \frac{1}{8+39.08{\left(\frac{D}{L}\right)}^{2}}=\frac{18\rho^{2}}{3\sqrt{3}\pi +78.16\rho }.$$ (20)

### 2.2 Calculation on the effective elastic modulus using the FE method

#### 2.2.1 Geometric model of the diamond structure

To calculate the effective elastic modulus of the diamond structure under loads in different directions and ensure the acceptable ranges of the approximate analytical solutions in the previous section, a series of models with porosities of 70%, 75%, 80%, 85%, and 90% were generated in the three loading directions of [001], [110], and [111], respectively. A 4 × 4 × 4 diamond lattice structure formed by cubic unit cells with an edge length of 2.0 mm was constructed, as shown in Figure 4d. The overall size of each model was 8.0 mm × 8.0 mm × 8.0 mm, and the number of unit cells had no significant effect on the stiffness of the whole lattice structure (Vijayavenkataraman et al., 2017).

#### 2.2.2 FE modeling

The diamond lattice model was placed in the x–y plane; a fixed hinge constraint was applied to the bottom surface that cannot move in the z-direction, and a load was applied to the top surface that can move downward at a constant velocity, compressing the height of the specimen by a strain of 10.0%, as shown in Figure 4d. The diamond structures were meshed using four-node tetrahedral elements. The mesh size was chosen as 0.05 mm after a convergence study. The elastic modulus of the diamond structure gradually converged as the mesh size decreased. The computational cost increased significantly with smaller mesh sizes. Therefore, to conserve computational resources, a mesh size of 0.05 mm was ultimately adopted. The material was Ti6Al4V, with an elastic modulus of 110.0 GPa and a Poisson’s ratio of 0.3. To further investigate the anisotropic mechanical properties of the diamond structures, the structures were gradually rotated about an axis in the [100] direction by 15° each time, and a series of loading directions that gradually changed from [001] to [010] were obtained. Similarly, the orientation dependencies in the (110) and (111) planes were investigated by modeling a range of other loading orientations. The mechanical properties of the diamond structure were calculated using ANSYS (v19.2, ANSYS Inc., Pittsburgh, Pennsylvania, United States of America).

### 2.3 Derivation of the effective elastic modulus using the theory of elasticity

#### 2.3.1 Basic theory of elasticity

In this part, some basic theory of elasticity was applied for calculating the effective elastic modulus of the diamond structure. There are nine stress constants acting on the cube element as shown in Equation 21 (Marc and Krishan, 2009):

$$\left[\begin{array}{ccc} \sigma_{11} & \sigma_{12} & \sigma_{13}\\ \sigma_{21} & \sigma_{22} & \sigma_{23}\\ \sigma_{31} & \sigma_{32} & \sigma_{33} \end{array}\right]=\left[\begin{array}{ccc} \sigma_{11} & \sigma_{12} & \sigma_{13}\\ \sigma_{12} & \sigma_{22} & \sigma_{23}\\ \sigma_{13} & \sigma_{23} & \sigma_{33} \end{array}\right],$$ (21)

where $\sigma_{13}=\sigma_{31}$ , $\sigma_{12}=\sigma_{21}$ , and $\sigma_{23}=\sigma_{32}$ (Marc and Krishan, 2009). When the cubic element is rotated, the stress state at that point remains the same, but the stress constants change. Similarly, the strain matrix is symmetric and provided in Equation 22 (Marc and Krishan, 2009):

$$\left[\begin{array}{ccc} \varepsilon_{11} & \varepsilon_{12} & \varepsilon_{13}\\ \varepsilon_{21} & \varepsilon_{22} & \varepsilon_{23}\\ \varepsilon_{31} & \varepsilon_{32} & \varepsilon_{33} \end{array}\right]=\left[\begin{array}{ccc} \varepsilon_{11} & \varepsilon_{12} & \varepsilon_{13}\\ \varepsilon_{12} & \varepsilon_{22} & \varepsilon_{23}\\ \varepsilon_{13} & \varepsilon_{23} & \varepsilon_{33} \end{array}\right].$$ (22)

The corner notations of the stresses and strains expressed in matrices are replaced by integers from 1 to 6, so the general cases of the stresses and strains are expressed as shown in Equations 23, 24, respectively (Marc and Krishan, 2009):

$$\sigma =\left[\begin{array}{ccc} \sigma_{1} & \sigma_{6} & \sigma_{5}\\ \sigma_{6} & \sigma_{2} & \sigma_{4}\\ \sigma_{5} & \sigma_{4} & \sigma_{3} \end{array}\right],$$ (23)

$$\varepsilon =\left[\begin{array}{ccc} \varepsilon_{1} & \varepsilon_{6}/2 & \varepsilon_{5}/2\\ \varepsilon_{6}/2 & \varepsilon_{2} & \varepsilon_{4}/2\\ \varepsilon_{5}/2 & \varepsilon_{4}/2 & \varepsilon_{3} \end{array}\right],$$ (24)

where $\varepsilon_{1}=\varepsilon_{11}$ , $\varepsilon_{2}=\varepsilon_{22}$ , $\varepsilon_{3}=\varepsilon_{33}$ , $\varepsilon_{4}=2\varepsilon_{23}=\gamma_{23}$ , $\varepsilon_{5}=2\varepsilon_{13}=\gamma_{13}$ , and $\varepsilon_{6}=2\varepsilon_{12}=\gamma_{12}$ . The differences in notations are important to maintain consistency in the equations for the relationship between stresses and strains.

Two elastic constants, ***C*** (stiffness) and ***S*** (compliance), can be used to relate stress components to strain components, i.e., $\sigma_{i}=C_{ij}\varepsilon_{j}$ and $\varepsilon_{j}=S_{ij}\sigma_{i}$ . The stress–strain relation is provided in Equation 25 (Marc and Krishan, 2009):

$$\left[\begin{array}{c} \begin{array}{c} \sigma_{1}\\ \sigma_{2}\\ \sigma_{3} \end{array}\\ \begin{array}{c} \sigma_{4}\\ \sigma_{5}\\ \sigma_{6} \end{array} \end{array}\right]=\left[\begin{array}{cc} \begin{array}{ccc} C_{11} & C_{12} & C_{13}\\ & C_{22} & C_{23}\\ & & C_{33} \end{array} & \begin{array}{ccc} C_{14} & C_{15} & C_{16}\\ C_{24} & C_{25} & C_{26}\\ C_{34} & C_{35} & C_{36} \end{array}\\ \text{symmetric} & \begin{array}{ccc} C_{44} & C_{45} & C_{46}\\ & C_{55} & C_{56}\\ & & C_{66} \end{array} \end{array}\right]\left[\begin{array}{c} \begin{array}{c} \varepsilon_{1}\\ \varepsilon_{2}\\ \varepsilon_{3} \end{array}\\ \begin{array}{c} \varepsilon_{4}\\ \varepsilon_{5}\\ \varepsilon_{6} \end{array} \end{array}\right].$$ (25)

The elastic stiffness and compliance matrices are symmetric, and there are only 21 independent components.

The new structure coincides exactly with the original structure when rotated 120° around the axis [1 1 1], where the basis vectors of the unit cell coordinate system are as follows (Equation 26):

$$e_{1}=\left[\begin{array}{c} 1\\ 0\\ 0 \end{array}\right],e_{2}=\left[\begin{array}{c} 0\\ 1\\ 0 \end{array}\right],\text{and }e_{3}=\left[\begin{array}{c} 0\\ 0\\ 1 \end{array}\right].$$ (26)

The basis vectors of the transformed coordinate system are expressed in Equation 27:

$$e_{1}^{\prime }=\left[\begin{array}{c} 0\\ 0\\ 1 \end{array}\right],e_{2}^{\prime }=\left[\begin{array}{c} 1\\ 0\\ 0 \end{array}\right],\text{and }e_{3}^{\prime }=\left[\begin{array}{c} 0\\ 1\\ 0 \end{array}\right].$$ (27)

The coordinate transformation matrix ***T*** is expressed in Equation 28:

$$T=\left[\begin{array}{ccc} 0 & 1 & 0\\ 0 & 0 & 1\\ 1 & 0 & 0 \end{array}\right].$$ (28)

***M*** and ***N*** can be calculated using matrix ***T*** as follows (Equations 29, 30):

$$M=\left[\begin{array}{cc} \begin{array}{ccc} \begin{array}{c} \begin{array}{c} 0\\ 0\\ 1 \end{array}\\ \begin{array}{c} 0\\ 0\\ 0 \end{array} \end{array} & \begin{array}{c} \begin{array}{c} 1\\ 0\\ 0 \end{array}\\ \begin{array}{c} 0\\ 0\\ 0 \end{array} \end{array} & \begin{array}{c} \begin{array}{c} 0\\ 1\\ 0 \end{array}\\ \begin{array}{c} 0\\ 0\\ 0 \end{array} \end{array} \end{array} & \begin{array}{ccc} \begin{array}{c} \begin{array}{c} 0\\ 0\\ 0 \end{array}\\ \begin{array}{c} 0\\ 0\\ 1 \end{array} \end{array} & \begin{array}{c} \begin{array}{c} 0\\ 0\\ 0 \end{array}\\ \begin{array}{c} 1\\ 0\\ 0 \end{array} \end{array} & \begin{array}{c} \begin{array}{c} 0\\ 0\\ 0 \end{array}\\ \begin{array}{c} 0\\ 1\\ 0 \end{array} \end{array} \end{array} \end{array}\right],$$ (29)

$$N=\left[\begin{array}{cc} \begin{array}{ccc} \begin{array}{c} \begin{array}{c} 0\\ 0\\ 1 \end{array}\\ \begin{array}{c} 0\\ 0\\ 0 \end{array} \end{array} & \begin{array}{c} \begin{array}{c} 1\\ 0\\ 0 \end{array}\\ \begin{array}{c} 0\\ 0\\ 0 \end{array} \end{array} & \begin{array}{c} \begin{array}{c} 0\\ 1\\ 0 \end{array}\\ \begin{array}{c} 0\\ 0\\ 0 \end{array} \end{array} \end{array} & \begin{array}{ccc} \begin{array}{c} \begin{array}{c} 0\\ 0\\ 0 \end{array}\\ \begin{array}{c} 0\\ 0\\ 1 \end{array} \end{array} & \begin{array}{c} \begin{array}{c} 0\\ 0\\ 0 \end{array}\\ \begin{array}{c} 1\\ 0\\ 0 \end{array} \end{array} & \begin{array}{c} \begin{array}{c} 0\\ 0\\ 0 \end{array}\\ \begin{array}{c} 0\\ 1\\ 0 \end{array} \end{array} \end{array} \end{array}\right].$$ (30)

Since the structure of diamond completely overlaps before and after the transformation, the following relation (Equation 31) exists:

$$C=C^{\prime }=NCM^{-1}.$$ (31)

Therefore, the equivalent stiffness matrix of the diamond structure can be simplified as shown in Equation 32:

$$C=\left[\begin{array}{cc} \begin{array}{ccc} C_{11} & C_{12} & C_{12}\\ C_{12} & C_{11} & C_{12}\\ C_{12} & C_{12} & C_{11} \end{array} & \begin{array}{ccc} C_{14} & C_{15} & C_{16}\\ C_{16} & C_{14} & C_{15}\\ C_{15} & C_{16} & C_{14} \end{array}\\ \begin{array}{ccc} C_{14} & C_{16} & C_{15}\\ C_{15} & C_{25} & C_{16}\\ C_{16} & C_{15} & C_{36} \end{array} & \begin{array}{ccc} C_{44} & C_{45} & C_{45}\\ C_{45} & C_{44} & C_{45}\\ C_{45} & C_{45} & C_{44} \end{array} \end{array}\right],$$ (32)

where there are seven independent elastic constants in Equation 32.

The expression for the diamond structure is provided in Equation 1. By applying mirror symmetry about the *YOZ* plane, the expression for the bone scaffold is presented in Equation 33:

$$\sin\left(-X\right)\sin\left(Y\right)\sin\left(Z\right)+\sin\left(-X\right)\cos\left(Y\right)\cos\left(Z\right)+\cos\left(-X\right)\sin\left(Y\right)\cos\left(Z\right)$$

$$+\cos\left(-X\right)\cos\left(Y\right)\sin\left(Z\right)-t\;>\;0.$$ (33)

The expression above can be transformed using trigonometric relations and is presented in Equation 34:

$$\sin\left(X-\frac{\pi }{2}\right)\sin\left(Y+\pi \right)\sin\left(Z-\frac{\pi }{2}\right)+\sin\left(X-\frac{\pi }{2}\right)\cos\left(Y+\pi \right)\cos\left(Z-\frac{\pi }{2}\right)$$

$$+\cos\left(X-\frac{\pi }{2}\right)\sin\left(Y+\pi \right)\cos\left(Z-\frac{\pi }{2}\right)+\cos\left(X-\frac{\pi }{2}\right)\cos\left(Y+\pi \right)\sin\left(Z-\frac{\pi }{2}\right)-t\;>\;0.$$ (34)

The equivalent stiffness matrix of the diamond structure can also be further simplified as presented in Equation 35:

$$C=\left[\begin{array}{cc} \begin{array}{ccc} C_{11} & C_{12} & C_{12}\\ C_{12} & C_{11} & C_{12}\\ C_{12} & C_{12} & C_{11} \end{array} & \\ & \begin{array}{ccc} C_{44} & & \\ & C_{44} & \\ & & C_{44} \end{array} \end{array}\right].$$ (35)

There are three independent elastic constants $C_{11},C_{12},\text{and }C_{44}$ in the cubic system.

For isotropic structures, the equation can be expressed as follows:

$$C_{44}=\frac{C_{11}-C_{12}}{2}.$$ (36)

For anisotropic structures, Equation 36 does not hold, and hence, the anisotropy ratio (Zener anisotropy index) is defined as follows (Equation 37):

$$A=\frac{2C_{44}}{C_{11}-C_{12}}.$$ (37)

For the elastic compliance matrix of the cubic system, the compliance matrix can be expressed as shown in Equation 38:

$$S=\left[\begin{array}{cc} \begin{array}{ccc} S_{11} & S_{12} & S_{12}\\ S_{12} & S_{11} & S_{12}\\ S_{12} & S_{12} & S_{11} \end{array} & \\ & \begin{array}{ccc} S_{44} & & \\ & S_{44} & \\ & & S_{44} \end{array} \end{array}\right].$$ (38)

The elastic modulus in any direction can be determined from $S_{11}$ , $S_{12}$ , and $S_{44}$ and is presented in Equation 39 (Kang et al., 2020):

$$\frac{1}{E}={\;S}_{11}\left(l_{1}^{4}+l_{2}^{4}+l_{3}^{4}\right)+\left(S_{44}+2S_{12}\right)\left[{\left(l_{2}l_{3}\right)}^{2}+{\left(l_{1}l_{3}\right)}^{2}+{\left(l_{1}l_{2}\right)}^{2}\right],$$ (39)

where $l_{1}$ , $l_{2}$ , and $l_{3}$ are the direction cosines in three orthogonal base directions. $S_{ij}$ represents the constants in the compliance matrix, which is the inverse matrix of the stiffness matrix. Using MATLAB (vR 2016a, MathWorks Inc., Natick, Massachusetts, United States of America), the 3D spatial distribution of the effective elastic modulus can be plotted.

#### 2.3.2 Boundary conditions for calculating the effective elastic modulus

**FIGURE 5:**
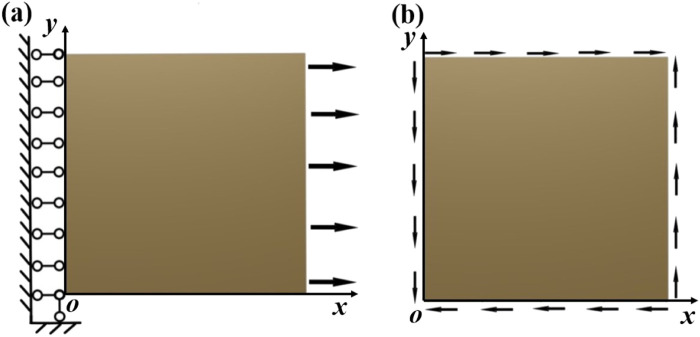
Boundary conditions. **(a)** For calculating ${\;S}_{11}$ and ${\;S}_{12}$ . **(b)** For calculating ${\;S}_{44}$ .

The boundary conditions for calculating the stiffness and compliance matrices of the structure are shown in Figure 5. As shown in Figure 5a, to calculate ${\;S}_{11}$ and ${\;S}_{12}$ in the compliance matrix of the structure, the following displacement boundary conditions were imposed on the unit cell of the structure with a length of *L*:

1. In the plane x=0 , $u_{x}=0$ , while on the plane x=L , the displacement is set to $u_{x}=0.01L$ .
2. In the planes y=0 and y=L , no constraints and loads are set.
3. In the planes z=0 and z=L , no constraints and loads are set.
4. At the point *O* (0,0,0), $u_{x}=0$ , $u_{y}=0$ , and $u_{z}=0$ .

Afterward, based on the reaction force generated on the plane x=0 and the calculated corresponding average stress $\sigma_{x}$ , the average relative displacements in the planes y=0 and y=L are calculated, and after calculating the strains in the *x*- and *y*-directions, ${\;S}_{11}$ and ${\;S}_{12}$ in the compliance matrix ***S*** are finally derived using Equation 40:

$$\left[\begin{array}{c} \begin{array}{c} \varepsilon_{1}\\ \varepsilon_{2}\\ \varepsilon_{3} \end{array}\\ \begin{array}{c} \varepsilon_{4}\\ \varepsilon_{5}\\ \varepsilon_{6} \end{array} \end{array}\right]=\left[\begin{array}{cc} \begin{array}{ccc} S_{11} & S_{12} & S_{12}\\ S_{12} & S_{11} & S_{12}\\ S_{12} & S_{12} & S_{11} \end{array} & \\ & \begin{array}{ccc} S_{44} & & \\ & S_{44} & \\ & & S_{44} \end{array} \end{array}\right]\left[\begin{array}{c} \begin{array}{c} \sigma_{1}\\ 0\\ 0 \end{array}\\ \begin{array}{c} 0\\ 0\\ 0 \end{array} \end{array}\right].$$ (40)

The two relevant constants can be obtained as shown in Equations 41, 42:

$$S_{11}=\frac{\varepsilon_{1}}{\sigma_{1}},$$ (41)

$$S_{12}=\frac{\varepsilon_{2}}{\sigma_{1}}.$$ (42)

As shown in Figure 5b, to calculate ${\;S}_{44}$ in the compliance matrix of the bone scaffold, the following displacement boundary conditions are imposed on the unit cell of the bone scaffold with a length of *L*:

1. In the *x*–*y* plane, γ=0.01 .
2. In the planes z=0 and z=L , no constraints and loads are set.

Similarly, ${\;S}_{44}$ in the compliance matrix ***S*** can be calculated using Equation 43:

$$\left[\begin{array}{c} \begin{array}{c} \varepsilon_{1}\\ \varepsilon_{2}\\ \varepsilon_{3} \end{array}\\ \begin{array}{c} \varepsilon_{4}\\ \varepsilon_{5}\\ \varepsilon_{6} \end{array} \end{array}\right]=\left[\begin{array}{cc} \begin{array}{ccc} S_{11} & S_{12} & S_{12}\\ S_{12} & S_{11} & S_{12}\\ S_{12} & S_{12} & S_{11} \end{array} & \\ & \begin{array}{ccc} S_{44} & & \\ & S_{44} & \\ & & S_{44} \end{array} \end{array}\right]\left[\begin{array}{c} \begin{array}{c} 0\\ 0\\ 0 \end{array}\\ \begin{array}{c} 0\\ 0\\ \sigma_{6} \end{array} \end{array}\right].$$ (43)

$S_{44}$ can be calculated using Equation 44:

$$S_{44}=\frac{\varepsilon_{6}}{\sigma_{6}}.$$ (44)

### 2.4 Experimental tests

To validate whether the results obtained from the aforementioned non-experimental methods are acceptable, quasi-static uniaxial compression tests were conducted. Samples were fabricated using additive manufacturing technology and loaded using a universal testing machine.

The diamond structures were fabricated using SLM (Renishaw AM400, Wotton-under-Edge, United Kingdom). Ti6Al4V powders were melted in a 99.99% argon atmosphere. The manipulation was performed using a laser power of 280 W and a scanning speed of 7.3 mm/sin this study. The samples had an edge length of 8.0 mm and a porosity of 80%. The samples were prepared by rotating around the [100] direction at 0°, 45°, and 90°. Additionally, samples with porosities of 75% and 70% were also prepared at 0°. In total, 15 samples were fabricated, with three samples prepared for each structure. Additionally, to demonstrate that other materials and printing techniques may also yield favorable results, polylactic acid (PLA) and fused deposition modeling (FDM) were also used to fabricate samples with a porosity of 70%. In particular, three samples were fabricated using PLA via FDM with a Bambu Lab A1 printer (Shenzhen, China). A 0.4 mm nozzle was used for printing, with a set printing speed of 125.0 mm/s and a fixed layer height of 0.1 mm. The $E_{s}$ of PLA was 2.58 GPa.

**FIGURE 6:**
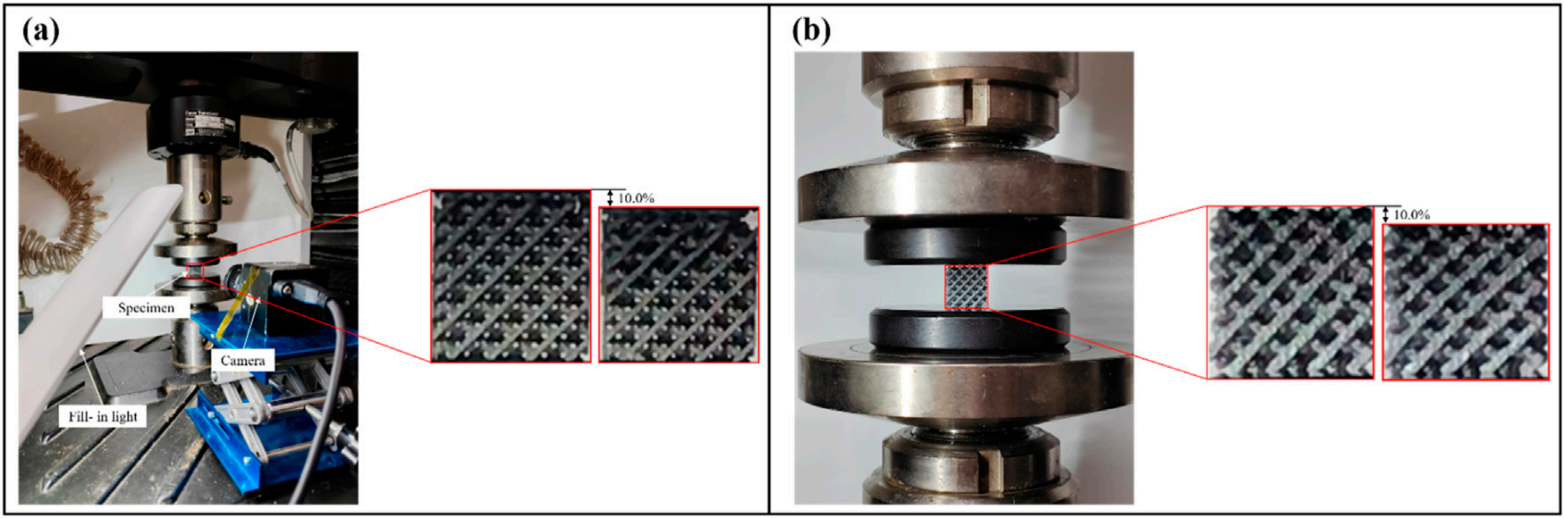
Experimental testing platform used for quasi-static uniaxial compressive tests. **(a)** Experimental test for the Ti6Al4V specimen. **(b)** Experimental test for the PLA specimen.

Quasi-static uniaxial compressive tests were performed using an MTS universal material testing machine (model: MTS Criterion 43.104). The loading speed was 0.1 mm/min. To ensure that the experimental tests fully encompassed the linear elastic phase, the specimens were subjected to a compressive strain of 10.0%, as shown in Figure 6. As the loading force was applied, the force and displacement data were recorded. The samples with different porosities were tested under the same loading conditions. The effective elastic modulus was calculated from the slope of the linear elastic part of the stress–strain curve.

In our previous study, TPMS scaffolds were manufactured via SLM and subsequently scanned using μ CT (Lu et al., 2020). Geometric models were reconstructed based on the μ CT images, and FE analyses were performed. The results indicated that the reconstructed models exhibited an increase in material volume of 68.1 ± 8.6%, and the effective elastic moduli of reconstructed models from FE analyses exceeded the experimental results by more than 50%. Although the experimentally measured effective elastic moduli of the scaffolds are lower than those of the original FE model due to incomplete bonding and partial melting of the powder, the differences between the two remain within 24.4%. The FE results of the original model are in closer agreement with the experimental results. Therefore, the FE results were used as the reference in the present work.

## 3 Results

### 3.1 Experimental validation

**FIGURE 7:**
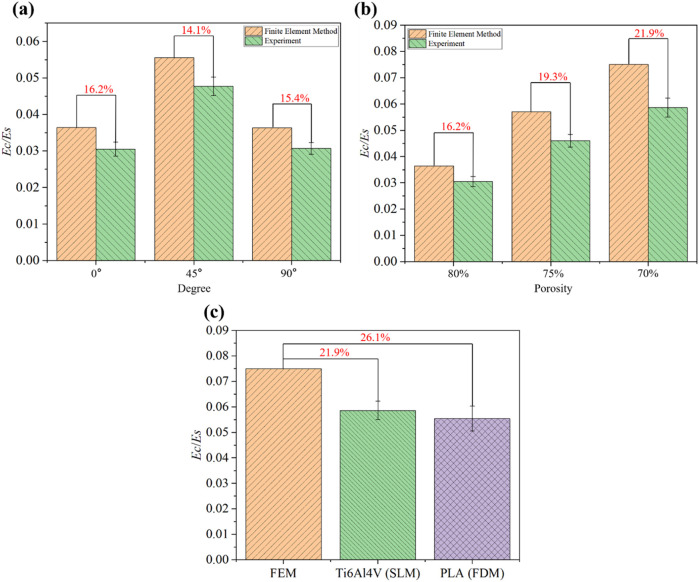
The dimensionless Young’s moduli of Diamond structures obtained from the FE method and experiment. **(a)** Different degrees rotate around the [100] direction at a porosity of 80%. **(b)** Different porosities at 0°. **(c)** Different materials and techniques at a porosity of 70%.

The non-experimental methods were validated through quasi-static uniaxial compression tests. The dimensionless Young’s modulus was calculated from the elastic modulus of the structure ( $E_{c}$ ) and the elastic modulus of the solid material ( $E_{s}$ ). The dimensionless Young’s modulus was lower at 0° and 90°, while it was higher at 45°, as shown in Figure 7a. As the structural porosity decreased, the dimensionless Young’s modulus decreased, as shown in Figure 7b. The FE results were used as a reference for comparison with the experimental results. It can be observed that the trend of experimental results was consistent with that of the FE results although the experimental results were lower. This discrepancy is likely due to differences between the CAD geometry and additively manufacturing scaffold geometry. Based on our previous work (Lu et al., 2020), experimental data were within admissible levels when the differences between experimental data and FE results are less than 30%, implying that the results (in Sections 3.2 and 3.3) from the non-experimental methods are acceptable. These results can be compared with the predictions from the approximate analytical approach. Additionally, to demonstrate that other materials and printing techniques may also yield favorable results, PLA material and FDM were used, as shown in Figure 7c. The experimental results from two materials and printing techniques are lower than the corresponding FE results, but both differences were less than 30%. The dimensionless Young’s modulus varied slightly using PLA material and FDM, with equally satisfactory results.

### 3.2 Comparison of the predicted porosity-related modulus with the results from FE and elasticity theory

**FIGURE 8:**
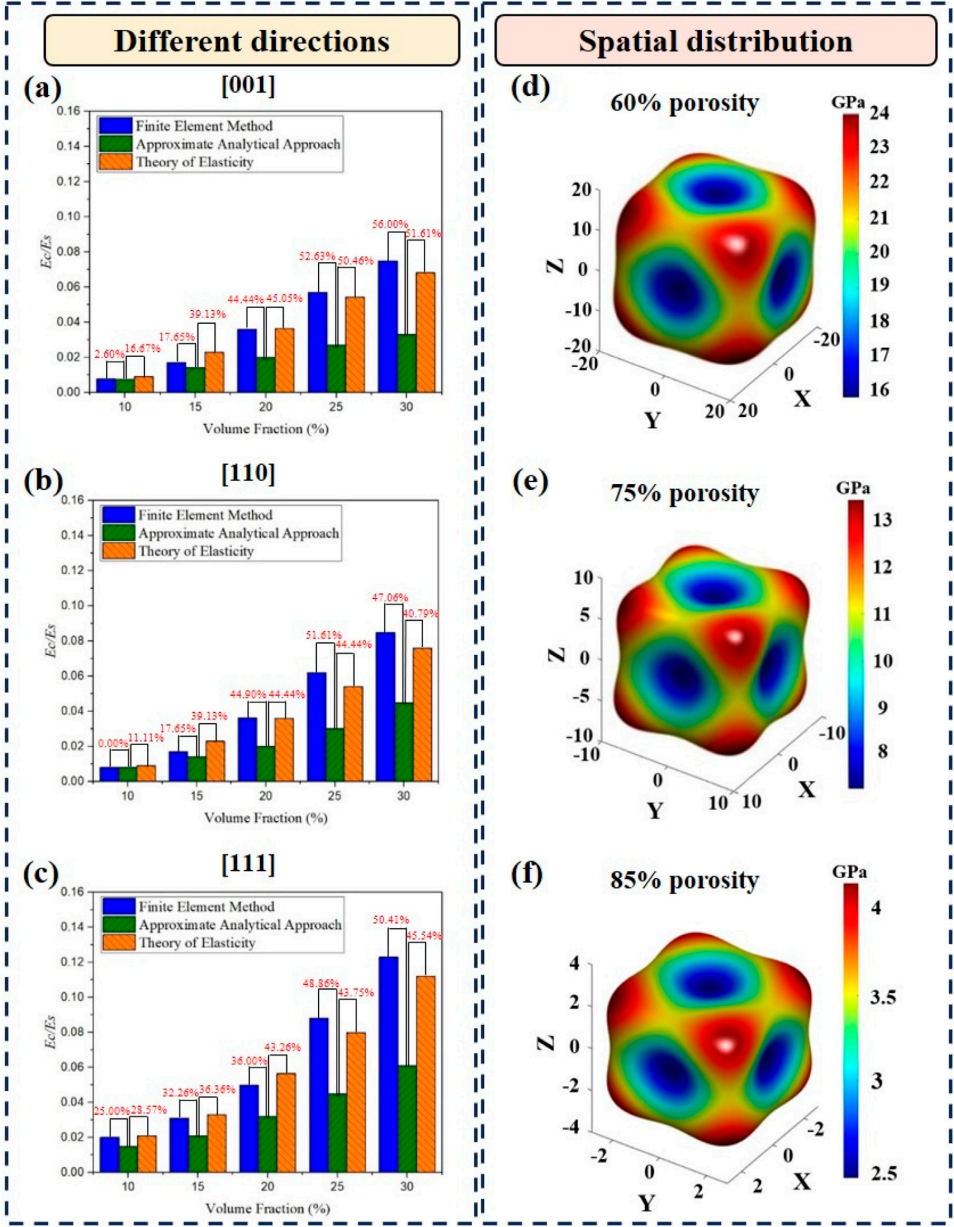
Dimensionless Young’s moduli of Diamond structures obtained from the FE method, the approximate analytical approach, and the theory of elasticity in **(a)** [001] direction, **(b)** [110] direction, and **(c)** [111] direction. The Young’s modulus surfaces of the Diamond structures with **(d)** 60%porosity, **(e)** 75% porosity, and (f) 85% porosity.

Comparing the three methods for calculating the effective elastic modulus of the diamond structure, the approximate analytical solutions were relatively closer to the FE results and elasticity theory at high porosities. The effective elastic modulus of the structure, $E_{c}$ , was calculated by substituting the elastic modulus of the solid material Ti6Al4V, $E_{s}=110.0$ GPa. The dimensionless Young’s modulus can be calculated from $E_{c}$ and $E_{s}$ . Approximate analytical solutions, FE results, and the results from the elasticity theory were compared, as shown in Figures 8a–c. In this study, the differences between the approximate analytical solutions and the results from the FE method and between the approximate analytical solutions and the results from elasticity theory did not exceed 30% and 40%, respectively, which are considered acceptable ranges. In previous studies, gyroid structures have been studied for their elastic moduli at different porosities and strut orientations (Yang et al., 2019). It is shown that, for the [111] direction, the consistency between analytical solutions and FE results is observed only when the structural porosity is higher than 90%. This is because the difference between their analytical solutions and the finite element results is approximately 30%, which was taken as the criterion in this article. Moreover, since elasticity theory is an ideal physical theory, the difference between the approximate analytical solution and the result from elasticity theory is larger than that between the approximate analytical solution and the result from the FE method. Therefore, the difference of 40% was considered the criterion for comparing the approximate analytical solution with the result from elasticity theory. For structural porosities below 85% (i.e., volume fractions higher than 15%), the differences are too large to be negligible due to simplification in modeling. As shown in Figures 8a,b, the differences between the approximate analytical solution and the result from the FE method and between the approximate analytical solution and the result from elasticity theory were 17.65% and 39.13%, respectively, when the volume fraction of the structure was 15%. Therefore, for structural porosity higher than 85%, the effective elastic modulus obtained from the approximate analytical method was closer to the FE solutions and the results from the elasticity theory in the [001] and [110] directions. However, there is a relatively large difference between approximate analytical solutions and results from two other methods in the [111] direction, as shown in Figure 8c. The differences between the approximate analytical solution and the result from the FE method and between the approximate analytical solution and the result from elasticity theory were 25.00% and 28.57%, respectively, when the volume fraction of the structure was 10%. Therefore, the approximate analytical method can be applied to calculate the effective elastic modulus in the [111] direction for the structural porosity higher than 90%. As the porosity increased, the effective elastic modulus of the structure gradually decreased, and the anisotropy was higher, as shown in Figures 8d–f. It can be observed that there was no distinct change in the shapes of the Young’s modulus surfaces for different porosities, indicating that the spatial distribution of the effective elastic modulus was not affected by the change in porosity.

### 3.3 Comparison of the predicted orientation-related modulus with the results from FE and elasticity theory

**FIGURE 9:**
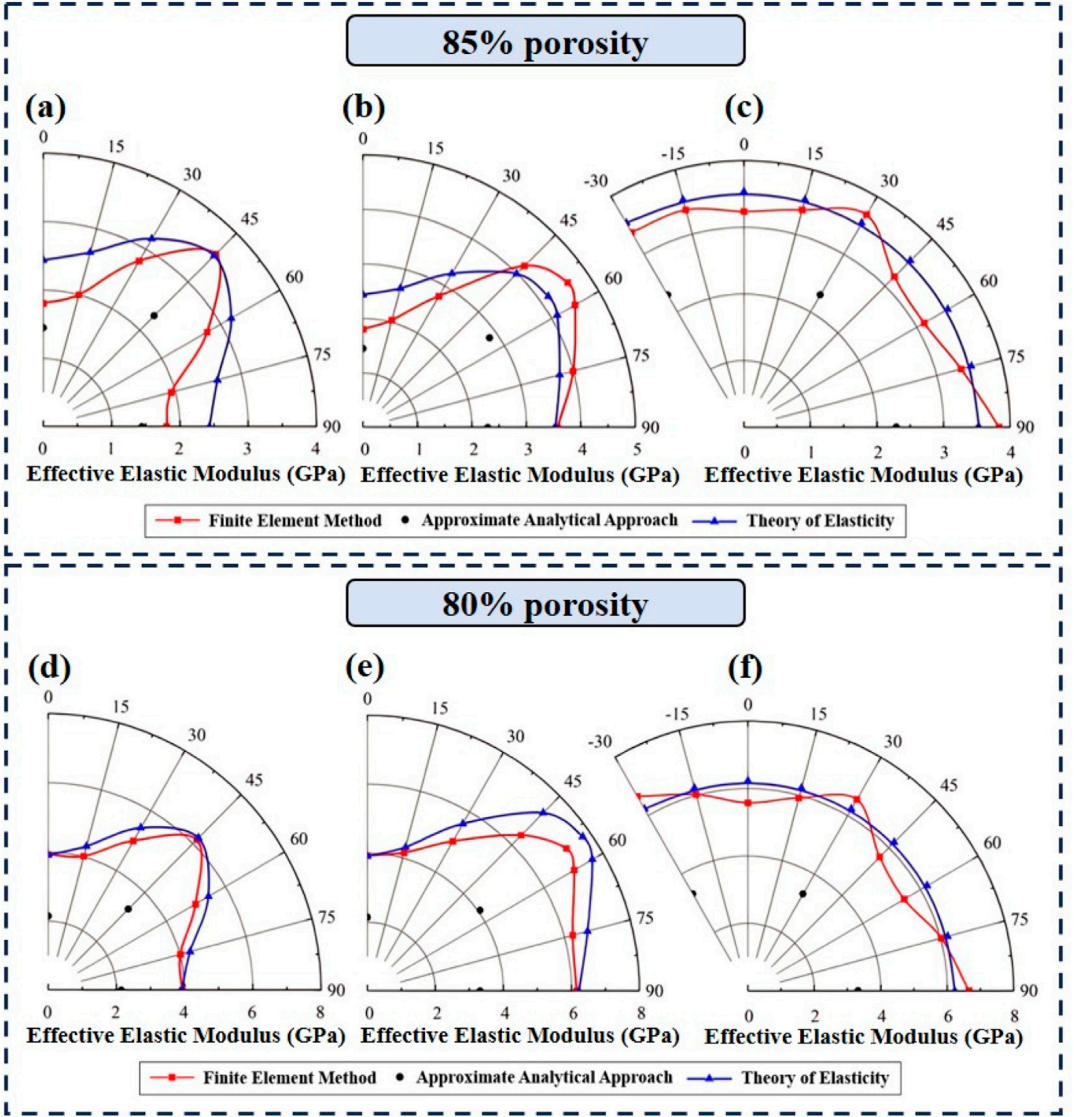
Polar diagrams of the effective elastic moduli obtained from the approximate analytical approach, the FE method, and the theory of elasticity for the Diamond structures **(a)** in (100) plane at 85% porosity, **(b)** in (110) plane at 85% porosity, **(c)** in (111) plane at 85% porosity, **(d)** in (100) plane at 80% porosity, **(e)** in (110) plane at 80% porosity, and **(f)** in (111) plane at 80% porosity.

At the same structural porosity, the difference in effective elastic moduli obtained from the three methods varied with the variation in structural orientation. The effective elastic moduli of the diamond structures with a porosity of 85% in the (100), (110), and (111) planes are shown in Figures 9a–c, respectively. The difference in the effective elastic modulus of the structures with the same porosity was caused only by the structural orientation. When the structural orientation was rotated along the [100] and [110] directions, there were different effective elastic moduli in different orientations. The effective elastic moduli varied significantly with the orientations in the (100) and (110) planes, whereas there was no significant variation in the effective elastic modulus in the (111) plane. The FE results agreed relatively well with those from the theory of elasticity, especially in the (111) plane. The trend of the approximate analytical solutions was basically consistent with that of the results from the theory of elasticity, and there was little change in the difference between the two methods when the rotation angle changed. In the (100) and (110) planes, the difference between the approximate analytical solutions and the FE results increased first and then decreased as the rotation angle changed from 0° to 90°. However, in the (111) plane, the differences between the approximate analytical solutions and the FE results were consistently large as the rotation angle changed from −30° to 90°. Therefore, in the (100) plane, the predicted results were acceptable when the structural orientation was close to 0° or 90°. In the (110) plane, the predicted results were acceptable when the structural orientation was close to 0°. In the (111) plane, whether the predicted results can be accepted or not was basically independent of the structural orientation but was dependent on the porosity of the structure.

The difference in the effective elastic moduli obtained using the three methods at a porosity of 80% was also similar to that at a porosity of 85%, as shown in Figures 9d–f. As shown in Figure 9, the difference between the approximate analytical solutions and the FE results became larger as the porosity decreased.

## 4 Discussion

In this study, the approximate analytical prediction of the elastic properties of diamond structures with varying porosities and strut orientations was investigated, and the FE method and theory of elasticity were compared with the approximate analytical approach. Additionally, experimental tests were performed to validate the feasibility of the non-experimental methods. The FE method, which is commonly used and well-established, was used to represent non-experimental methods and was compared with experimental results. Therefore, the approximate analytical solutions and the results from elasticity theory were indirectly compared with experimental results. By changing the porosity and orientation of the structure, the range of different effective elastic moduli was obtained. The results from this study can serve as a reference for applying the approximate analytical approach to efficiently determine the structural elastic modulus. Two interesting findings were revealed in the present study.

First, the approximate analytical solutions were close to the results from FE and the elasticity theory for structures with porosity higher than 85%. In previous studies, the gyroid structures have been studied for their elastic moduli at different porosities and orientations (Yang et al., 2019). A comparison among the different methods revealed that the analytical solution was reasonable at solid volume fractions less than 20% (Yang et al., 2019). These conclusions are consistent with those obtained from the present work. Since the effective elastic modulus was calculated in the same way for regular and irregular porous structures, the conclusions obtained were of broad applicability. Moreover, errors may occur in the simplification of the TPMS structure into struts when using the approximate analytical approach for calculation. In low porosities, such a simplification can cause the model to deviate significantly from the original structure, especially at the nodal locations. In high porosities, the TPMS model was closer to the structure composed of struts. Therefore, the approximate analytical solutions were close to the results from the FE and elasticity theory at high porosities. Moreover, many assumptions were required in the theoretical calculations, which may result in some deviations from reality. Prior research has already used approximate analytical approaches to evaluate the elastic behavior of TPMS structures, such as the gyroid (Yang et al., 2019). However, due to different topologies of TPMS structures, there are some discrepancies among their results. The conclusions on gyroid structures cannot be directly applied to other TPMS structures. Therefore, it is necessary to have a systematic study on diamond structures using approximate analytical approaches.

Second, the difference in the effective elastic moduli obtained from the three methods changed with the variation in structural orientations at the same porosity, which may be caused by anisotropy in the structure. Therefore, the change in the angle led to some differences in the effective elastic moduli calculated from the three methods. Kang et al. (2020) used elasticity theory to demonstrate that the structures were highly anisotropic. Khaleghi et al. (2021) also reported the anisotropies of seven TPMS structures, such as elastic modulus, shear modulus, and Poisson’s ratio, which were also consistent with the results presented in this study. Moreover, our previous work also demonstrated the anisotropic characteristic of TPMS structures (Huo et al., 2024). The anisotropic characteristics of these microstructures are summarized in Table 1. Additionally, as the structural orientation was changed, the approximate analytical solution differed considerably from the results from the FE and elasticity theory, especially in the (111) plane. This may be caused by changes in the way forces are applied to each strut at different rotational angles, which can significantly impact the approximate analytical solutions. However, in the [001] and [110] directions, the approximate analytical solutions have an acceptable range compared to the solutions in the [111] direction. In the (001) and (110) planes, it can be clearly observed that the elastic moduli varied greatly with the angles of rotation, whereas the elastic modulus changed very little in the (111) plane. This phenomenon may be attributed to the cubic symmetry of the diamond structure, which results in minimal changes in the effective elastic modulus along the diagonal direction. Therefore, at the same porosity, in the (100) plane, the predicted results were acceptable when the structural orientation was close to 0° or 90°. In the (110) plane, the predicted results were acceptable when the structural orientation was close to 0°. In the (111) plane, the acceptability of the predicted results was basically independent of the structural orientation but depended on the porosity of the structure. Additionally, to demonstrate that other materials and printing techniques may also yield favorable results, PLA and FDM were also used to fabricate the samples with a porosity of 70%. The porosity and strut orientation of samples with varying materials and printing techniques were the same. It was shown that the results with dimensionless Young’s modulus are suitable for other materials and printing technologies.

**TABLE 1:** Summary of microstructures and their anisotropy.

Type of microstructures	Method	Result	Reference
Cubic spherical hollow unit, orthogonal cubic unit, body-centered cubic unit, and reinforced body-centered cubic unit	Finite element method and elasticity theory	The ratio of the highest modulus to the lowest for all porous structures is within a range of 1.6–2.4	Kang et al. (2020)
Schwarz-P, IWP, gyroid, diamond, FKS, FRD, and Neovius	Finite element method	Among the seven structures investigated, FKS showed the lowest universal anisotropy index for the range of 10%–90% of volume fraction for the solid phase. On the other hand, in the range of 10%–40% and 40%–90% of the volume fraction for the solid phase, IWP and diamond showed the highest universal anisotropy index, respectively	Khaleghi et al. (2021)
Schwarz-P and its variants	Finite element method and experimental test	With an increase in the radii of the multi-functional pores, the Zener anisotropy indices of the structures demonstrated an increasing trend, while the elastic moduli displayed a decreasing trend	Jiang et al. (2024)

In this study, the applicable range of the approximate analytical approach was identified based on different conditions when evaluating the mechanical properties of diamond structures. The calculation method chosen should be both accurate and efficient. The approximate analytical approach may be an excellent choice to predict structural properties at high porosity. The approximate analytical approach was simple and convenient, but it had a small range of applications due to model simplification. It should be noted that the purpose of the present study was to explore the applicable range of the approximate analytical approach, not to develop new methods. Therefore, the simplification in the approximate analytical method was chosen from that existing in the literature (Yang et al., 2019). The model can be discretized into small elements using the FE method. Thus, the error can be reduced, and the model approaches the actual condition more closely. However, the process of modeling and numerical simulation was complex and time-consuming. The calculation method of the elasticity theory was relatively fast, but the derivation of the equations was complex and required many assumptions. Therefore, the present study may help accurately and efficiently predict the mechanical properties of TPMS structures before formal calculations and experiments. From a purely mechanical or numerical analysis perspective, a difference of 30% is indeed substantial. However, during the initial design and screening stage of porous biological scaffolds, such differences are considered acceptable in practical engineering applications. When conducting preliminary screening among many possible structure–porosity combinations, designers require a rapid estimation method capable of determining the order of magnitude. A model that can control the difference in elastic modulus calculated between the approximate analytical approach and the FE method within 30% is sufficient to effectively distinguish between suitable and unsuitable design options. This enables the rapid elimination of numerous unsuitable candidates, allowing computational resources to be concentrated on more promising candidate designs.

Some limitations in the present work should be noted. First, to obtain the applicable range of the approximate analytical approach, only the diamond structure was investigated. However, TPMS scaffolds exhibit a wide variety of topologies. Among different TPMS structures, diamond was selected in this study because of its excellent mechanical properties (Wang et al., 2022; Kladovasilakis et al., 2021; Sokollu et al., 2022; Chen et al., 2019; Al-Ketan et al., 2018), such as high energy absorption and high ultimate tensile strength. Meanwhile, research on other TPMS structures is also of great significance. Second, only the elastic modulus, one of the commonly used mechanical properties for bone scaffolds, was calculated at different porosities and orientations of the structures in this study. More mechanical properties and other impact factors could also be explored in depth, such as shear modulus and Poisson’s ratio. Finally, it should be noted that the cylindrical strut in the analytical approach may be oversimplified, and the diamond structure loses connectivity at approximately 92% porosity. Therefore, the range of predictable porosity is relatively narrow. Simplifying the diamond structure using a more complex geometric model, such as conical shapes, may be more helpful in expanding the prediction range of the analytical method, which requires further investigations in the future.

## 5 Conclusion

In this study, approximate analytical prediction of the elastic properties of diamond structures with varying porosities and strut orientations was investigated, and the FE method and theory of elasticity were compared with the approximate analytical approach. Additionally, experimental tests were performed to validate the feasibility of the non-experimental methods. The main conclusions are listed as follows:

1. The effective elastic moduli obtained from the approximate analytical approach were closer to the results from FE and elasticity theory for structures with a porosity higher than 85% in the [001] and [110] directions, along with 90% in the [111] direction.
2. At the same porosity, in the (100) plane, the predicted results were acceptable when the structural orientation was close to 0° or 90°. In the (110) plane, the predicted results were acceptable when the structural orientation was close to 0°. In the (111) plane, whether the predicted results can be accepted was basically independent of the structural orientation, but it depended on the porosity of the structure.

The results obtained from this study can help engineers/researchers in applying the approximate analytical approach to simply and efficiently predict the mechanical properties of TPMS structures prior to performing formal calculations and experiments.

## Data Availability

The original contributions presented in the study are included in the article/supplementary material; further inquiries can be directed to the corresponding author.
